# Supplementary material for: Antibacterial and Synergistic Effects of Terminalia citrina Leaf Extracts Against Gastrointestinal Pathogens: Insights from Metabolomic Analysis
Source: Antibiotics (Basel). 2025 Jun 8;14(6):593. doi: 10.3390/antibiotics14060593 (PMC12189912; doi:10.3390/antibiotics14060593)
Supplement: Supplementary file 1 [file antibiotics-14-00593-s001.zip › antibiotics-3651380-supplementary.pdf]

antibiotics

MDPI

Supplementary Data

Antibacterial and Synergistic Effects of Terminalia citrina Leaf Extracts Against Gastrointestinal Pathogens: Insights from Metabolomic Analysis

Sze-Tieng Ang [1], Tak Hyun Kim [1], Matthew James Cheesman [2,*] and Ian Edwin Cock [1,*]

1 School of Environmental and Science, Griffith University, Nathan Campus, Brisbane 4111, Australia; k.ang@griffith.edu.au (S.-T.A.); t.kim@griffith.edu.au (T.H.K.)
2 School of Pharmacy and Medical Sciences, Griffith University, Gold Coast Campus, Gold Coast 4222, Australia
* Correspondence: m.cheesman@griffith.edu.au (M.J.C.); Tel.: +61-7-55529230 (M.J.C.); i.cock@griffith.edu.au (I.E.C.); Tel.: +61-7-3735-7637 (I.E.C.)

A

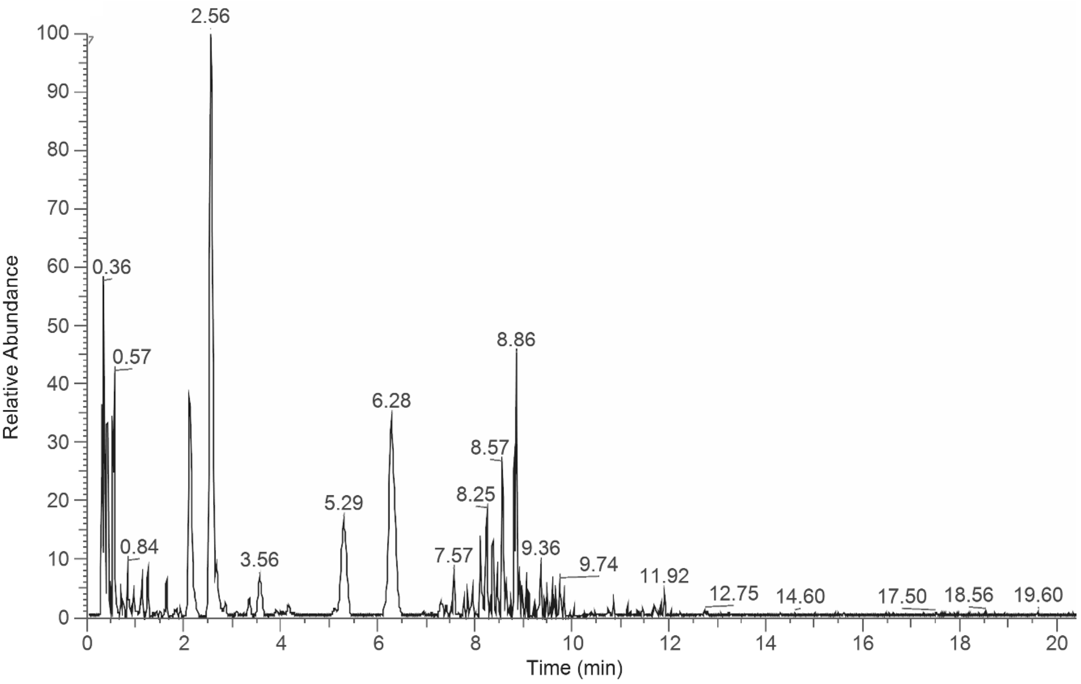

B

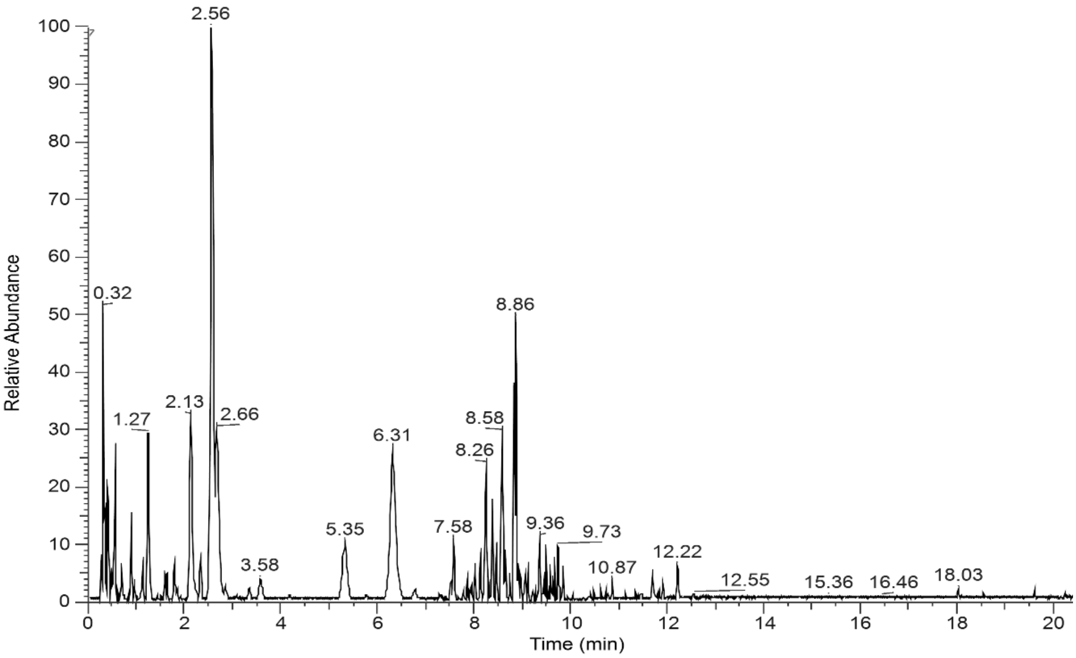

Figure S1: Total compound chromatograms in negative-ion mode for the (A) TciW and (B) TciM extracts.

A

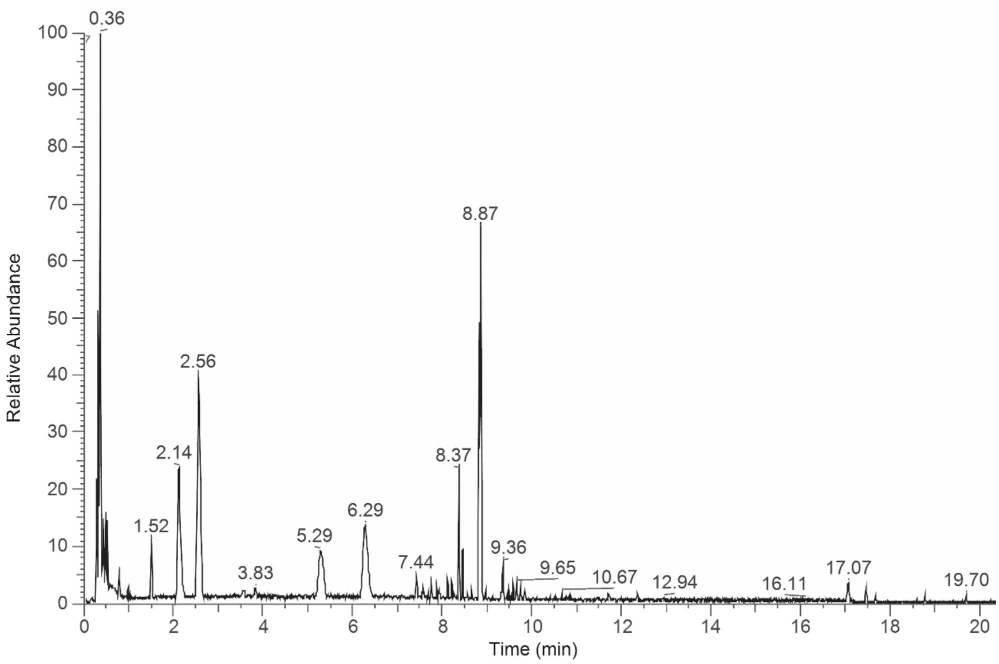

B

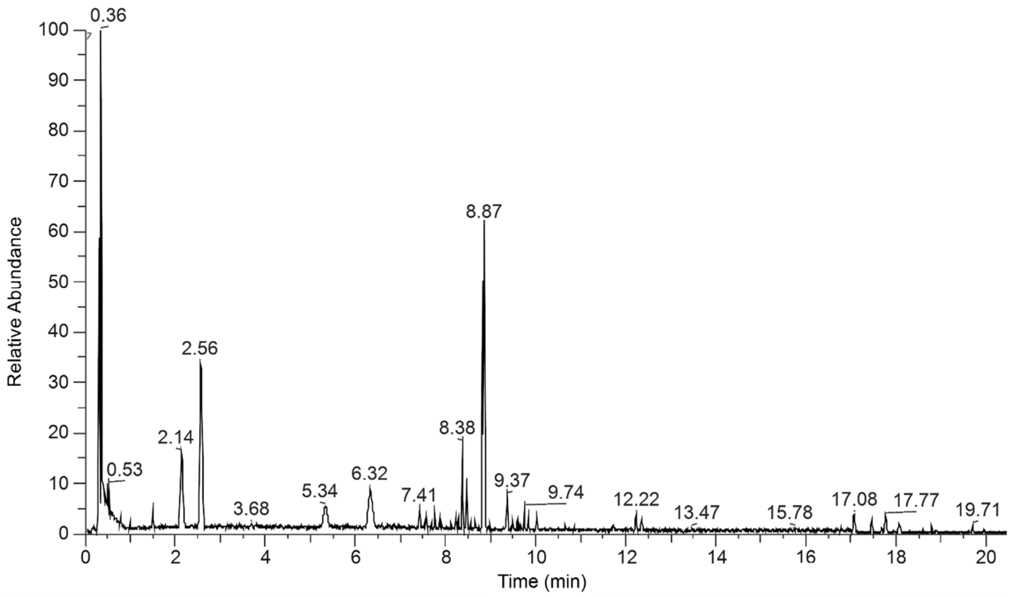

Figure S2. Total compound chromatograms in positive-ion mode of the (a) TciW and (b) TciM extracts.

Table S1. UPLC-MS putative identification and % relative abundance of polyphenols identified at positive-ion ([M+H]+1) mode in extracts of *Terminalia citrina*.

Rt (min)	Putative Identification	Empirical Formula	Molecular Weight	Relative Abundance (% Total Area)	
				TciW	TciM
7.029	(-)-Fustin	$C_{15}H_{12}O_6$	288.063	0.01	-
8.043	Apigetrin	$C_{21}H_{20}O_{10}$	432.105	0.016	0.013
8.123	Apigenin 7- (6''-p-coumarylglucoside)	$C_{30}H_{26}O_{12}$	578.14189	0.02	0.77
8.226	Dihydrokaempferol	$C_{15}H_{12}O_6$	288.063	T	0.03
8.376	(1ξ)-1,5-Anhydro-1-[2-(3,4-dihydroxyphenyl)-5,7-dihydroxy-4-oxo-4H-chromen-8-yl]-D-galactitol	$C_{21}H_{20}O_{11}$	448.10038	0.47	0.45
8.853	Chalconaringenin	$C_{15}H_{12}O_5$	272.06816	-	0.03
8.864	NP-018730	$C_{21}H_{20}O_{10}$	432.10538	1.89	2.09
9.365	Quercetin 3- (6''-p-hydroxybenzoylgalactoside)	$C_{28}H_{24}O_{14}$	584.11644	-	0.28
9.5	Pelargonidin	$C_{15}H_{10}O_5$	270.0526	T	0.02
9.616	Genistin	$C_{21}H_{20}O_{10}$	432.105	-	0.01
9.73	6-O-[(2E)-3-Phenyl-2-propenoyl]-1-O-(3,4,5-trihydroxybenzoyl)-β-D-glucopyranose	$C_{22}H_{22}O_{11}$	462.116	T	0.015
9.826	Aurasperone C	$C_{31}H_{28}O_{12}$	592.158	0.014	0.014
9.837	4-Hydroxy-3-(3-methyl-2-buten-1-yl)phenyl 6-O-[(2R,3R,4R)-3,4-dihydroxy-4-(hydroxymethyl)tetrahydro-2-furanyl]-beta-D-glucopyranoside	$C_{22}H_{32}O_{11}$	494.176	0.018	T
10.224	Vitexin 2''-p-hydroxybenzoate	$C_{28}H_{24}O_{12}$	552.127	0.01	0.016
10.271	NP-015285	$C_{21}H_{20}O_9$	416.11076	0.02	-
10.482	NP-018731	$C_{21}H_{22}O_{10}$	446.12114	0.02	0.02
10.67	Dichotosinin	$C_{24}H_{30}O_{10}$	478.184	0.04	-
10.852	Vitexin 2''-O- (E) -ferulate	$C_{31}H_{28}O_{13}$	608.15297	-	0.03
11.02	Glycitin	$C_{22}H_{22}O_{10}$	446.121	-	0.01

Compounds less than 0.01% of the total area were considered as trace amounts and denoted as T.

Table S2. UPLC-MS putative identification and % relative abundance of phytochemicals identified at both positive- ([M+H]+1) and negative-ion modes ([M-H]-1) in extracts of *Terminalia citrina*.

Rt (min)	Putative Identification	Empirical Formula	Molecular Weight	Relative Abundance (% Total Area)	
				TciW	TciM
0.313	Choline	$C_5 H_{13} N O$	103.1	0.86	1.08
0.315	L-Pyroglutamic acid	$C_5 H_7 N O_3$	129.042	0.06	0.04
0.32	Crotonic acid	$C_4 H_6 O_2$	86.0367	0.12	-
0.32	N-Acetylglucosaminitol	$C_8 H_{17} N O_6$	223.105	0.016	-
0.321	D-Mannoheptulose	$C_7 H_{14} O_7$	210.074	-	0.03
0.321	Gluconic acid	$C_6 H_{12} O_7$	196.058	0.57	0.17
0.321	Methyl (5R)-5-[(1S)-2-acetamido-1-hydroxyethyl]-alpha-D-lyxopyranoside	$C_{10} H_{19} N O_7$	265.116	0.48	-
0.322	3-Aminoaspartic acid	$C_4 H_8 N_2 O_4$	148.048	0.18	-
0.325	Pyruvic acid	$C_3 H_4 O_3$	88.0159	0.07	-
0.327	Hexaric acid	$C_6 H_{10} O_8$	210.037	0.09	-
0.328	2-(alpha-D-mannosyl)-D-glyceric acid	$C_9 H_{16} O_9$	268.079	0.13	-
0.329	Proline	$C_5 H_9 N O_2$	115.063	-	0.67
0.329	δ-Ribono-1,4-lactone	$C_5 H_8 O_5$	148.037	0.07	-
0.33	Glutaric anhydride	$C_5 H_6 O_3$	114.031	T	0.04
0.331	DL-Lactic Acid	$C_3 H_6 O_3$	90.0315	0.11	0.08
0.332	Aminoadipate	$C_6 H_9 N O_4$	159.053	0.01	-
0.333	D-(-)-Quinic acid	$C_7 H_{12} O_6$	192.063	-	0.19
0.335	D-(+)-Maltose	$C_{12} H_{22} O_{11}$	388.121	-	0.04
0.344	6-Aminocaproic acid	$C_6 H_{13} N O_2$	131.094	-	0.08
0.347	Ascorbic acid	$C_6 H_8 O_6$	176.032		0.01
0.348	g-Guanidinobutyrate	$C_5 H_{11} N_3 O_2$	145.085	-	0.03
0.348	Hopantenic acid	$C_{10} H_{19} N O_5$	233.126	0.02	-
0.351	4-Guanidinobutyric acid	$C_5 H_{11} N_3 O_2$	145.085	0.03	-
0.356	4-Oxoproline	$C_5 H_7 N O_3$	129.042	0.07	0.07
0.358	L-Norvaline	$C_5 H_{11} N O_2$	117.079	-	0.17
0.359	D-(+)-Malic acid	$C4 H_6 O_5$	134.021	0.89	0.37
0.359	Fumaric acid	$C_4 H_4 O_4$	116.011	0.34	0.13
0.365	Cyanoacetic acid	$C_3 H_3 N O_2$	85.0163	0.06	-
0.366	Pipecolic acid	$C_6 H_{11} N O_2$	129.079	2.13	-
0.374	Malonic acid	$C_3 H_4 O_4$	104.011	T	0.06
0.412	Dioxolide A	$C_9 H_{11} N O_4$	197.068	0.01	-
0.415	Chebulic acid	$C_{14} H_{12} O_{11}$	356.038	0.03	0.03

0.427	Dehydroascorbic acid	$C_6 H_6 O_6$	174.016	-	0.01
0.428	Clavulanic acid	$C_8 H_9 N O_5$	199.048	0.02	0.05
0.428	Uridine	$C_9 H_{12} N_2 O_6$	244.069	T	0.02
0.435	L-Tyrosine	$C_9 H_{11} N O_3$	181.074	0.06	0.07
0.442	Adenine	$C_5 H_5 N_5$	135.054	0.03	-
0.442	Adenosine	$C_{10} H_{13} N_5 O_4$	267.096	0.22	-
0.46	6-O-Galloyl-glucose	$C_{13} H_{16} O_{10}$	332.074	0.02	0.16
0.467	Acetophenone	$C_8 H_8 O$	120.057	0.04	0.06
0.497	(+/-)-2-Hydroxyglutaric acid	$C_5 H_8 O_5$	148.037	0.01	T
0.525	Isoleucine	$C_6 H_{13} N O_2$	131.094	-	0.26
0.534	L-(+)-Leucine	$C_6 H_{13} N O_2$	131.094	0.16	T
0.562	1-Salicylate glucuronide	$C_{13} H_{14} O_9$	314.063	0.05	0.09
0.568	Gallic acid	$C_7 H_6 O_5$	170.021	-	0.32
0.569	Pyrogallol	$C_6 H_6 O_3$	126.032	-	0.07
0.662	4-hydroxymethyl-2-methyl-3-furanylcarbonyl α-L-rhamnopyranoside	$C_{13} H_{18} O_8$	302.1	0.01	-
0.726	Corilagin	$C_{27} H_{22} O_{18}$	634.081	0.04	0.14
0.781	Sesbanimide A	$C_{15} H_{21} N O_7$	327.132	0.03	-
0.871	NP-020139	$C_{13} H_{16} O_9$	316.079	0.05	0.03
0.946	Koaburaside	$C_{14} H_{20} O_9$	332.111	0.01	0.01
0.979	5,7-Dihydroxy-4-oxo-4H-chromen-3-yl beta-D-xylopyranoside	$C_{14} H_{14} O_9$	326.064	-	0.01
0.981	Fertaric acid	$C_{14} H_{14} O_9$	326.064	0.01	-
0.986	Xanthurenic acid	$C_{10} H_7 N O_4$	205.037	0.08	0.06
1.017	Pantothenic acid	$C_9 H_{17} N O_5$	219.111	0.03	0.03
1.094	Gentisic acid	$C_7 H_6 O_4$	154.026	0.03	T
1.232	Kanzakiflavone 1	$C_{17} H_{12} O_7$	328.058	0.01	T
1.368	2'-alpha-mannosyl-L-tryptophan	$C_{17} H_{22} N_2 O_7$	366.142	0.02	-
1.506	4-Indolecarbaldehyde	$C_9 H_7 N O$	145.053	0.04	0.02
1.506	trans-3-Indoleacrylic acid	$C_{11} H_9 N O_2$	187.063	0.38	0.18
2.094	Kynurenic acid	$C_{10} H_7 N O_3$	189.042	0.06	0.06
2.137	Epigallocatechin 3-O- (4-hydroxybenzoate)	$C_{22} H_{18} O_9$	426.095	T	0.02
2.15	Alcanivorone	$C_{10} H_{10} O_5$	210.053	0.02	0.02
2.218	1,6-Bis-O-(3,4,5-trihydroxybenzoyl)hexopyranose	$C_{20} H_{20} O_{14}$	484.085	0.06	0.02
2.567	Catechin	$C_{15} H_{14} O_6$	290.079	9.80	7.31

2.936	4-Hydroxyphenyl 4-O-(3,4,5-trihydroxybenzoyl)-beta-D-glucopyranoside	$C_{19}H_{20}O_{11}$	424.101	-	0.03
3.06	1-O-(2-Hydroxybenzoyl)-D-glucopyranuronic acid	$C_{13}H_{14}O_{9}$	314.063	0.07	0.07
3.122	Dendromoniliside C	$C_{21}H_{32}O_{10}$	444.2	0.03	0.02
3.83	9-Aminononanoic acid	$C_{9}H_{19}NO_{2}$	173.142	0.19	0.14
4.072	Vitexin 2''-O-p-coumarate	$C_{30}H_{26}O_{12}$	578.143	2.31	0.55
4.159	1,2-Dimethyl-4-oxo-1,4-dihydro-3-pyridinyl beta-D-glucopyranoside	$C_{13}H_{19}NO_{7}$	301.116	0.02	T
5.158	5-O-a-D-glucopyranosyl-5-hydroxymellein	$C_{16}H_{20}O_{9}$	356.111	-	0.04
5.334	Apigenin 7- (6''-p-coumarylglucoside)	$C_{30}H_{26}O_{12}$	578.142	0.02	0.77
5.508	3,5-Dihydroxy-2-(4-hydroxyphenyl)-4-oxo-3,4-dihydro-2H-chromen-7-yl hexopyranoside	$C_{21}H_{22}O_{11}$	450.116	0.01	0.07
5.93	1,3,6-Trihydroxy-7-methoxy-9-oxo-9H-xanthen-2-yl beta-D-glucopyranoside	$C_{20}H_{20}O_{12}$	452.096	-	0.03
6.459	Benzyl Î²-primeveroside	$C_{18}H_{26}O_{10}$	402.152	0.05	0.04
6.69	sinapoylglucose	$C_{17}H_{22}O_{10}$	386.121	0.02	0.03
6.991	Eschweilenol A	$C_{20}H_{10}O_{11}$	426.022	T	0.02
7.029	(-)-Fustin	$C_{15}H_{12}O_{6}$	288.063	0.01	-
7.289	Acertannin	$C_{20}H_{20}O_{13}$	468.09	0.06	0.05
7.516	Ampelopsin 3'-glucoside	$C_{21}H_{22}O_{13}$	482.106	0.04	0.06
7.581	2-Acetamido-4-O-[3-(benzoylamino)-3-deoxy-beta-D-galactopyranosyl]-2-deoxy-D-glucopyranose	$C_{21}H_{30}N_{2}O_{11}$	486.185	-	0.01
7.585	Chlorogenic acid	$C_{16}H_{18}O_{9}$	336.082	0.01	-
7.624	12-O-Î²-D-Glucopyranosyloxyjasmonic acid	$C_{18}H_{28}O_{9}$	388.173	0.01	T
7.673	(1S,3S)-3-Glycoloyl-3,5,12-trihydroxy-10-methoxy-6,11-dioxo-1,2,3,4,6,11-hexahydro-1-tetracenyl 2,3,6-trideoxy-3-[(phenylacetyl)amino]hexopyranoside	$C_{35}H_{35}NO_{12}$	661.215	0.02	-
7.895	(-)-8-hydroxyjasmonic acid	$C_{12}H_{18}O_{4}$	226.12	0.01	0.01
8.024	Oosporein	$C_{14}H_{10}O_{8}$	306.037	-	0.01
8.027	1,3,6-Trigalloyl glucose	$C_{27}H_{24}O_{18}$	636.096	0.02	0.13
8.043	Apigetrin	$C_{21}H_{20}O_{10}$	432.105	0.02	0.01

8.124	Ixoside	$C_{16}H_{20}O_{11}$	388.1	0.01	-
8.13	Robinetinidol-(4alpha->8)-catechin-(6->4alpha)-robinetinidol	$C_{45}H_{38}O_{18}$	866.206	0.10	0.18
8.136	NP-021797	$C_{12}H_{22}O_3$	231.183	0.02	0.02
8.153	Terminalin	$C_{28}H_{10}O_{16}$	601.997	0.18	T
8.226	Dihydrokaempferol	$C_{15}H_{12}O_6$	288.063	T	0.03
8.276	Procyanidin B3 3-O-gallate	$C_{37}H_{30}O_{16}$	730.153	0.08	0.19
8.355	Phenethyl Î²-primeveroside	$C_{19}H_{28}O_{10}$	416.169	-	0.01
8.379	(1ξ)-1,5-Anhydro-1-[2-(3,4-dihydroxyphenyl)-5,7-dihydroxy-4-oxo-4H-chromen-8-yl]-D-galactitol	$C_{21}H_{20}O_{11}$	448.1	0.47	0.45
8.464	Orientin	$C_{21}H_{20}O_{11}$	448.1	0.18	0.20
8.579	Ellagic acid	$C_{14}H_6O_8$	302.006	0.998	0.98
8.592	NP-016596	$C_{19}H_{30}O_8$	432.199	-	0.03
8.644	3-Hydroxy-5-[(E)-2-(4-hydroxyphenyl)vinyl]phenyl 6-O-beta-D-glucopyranosyl-beta-L-glucopyranoside	$C_{26}H_{32}O_{13}$	552.184	0.02	0.02
8.843	Lariciresinol 4-O-glucoside	$C_{26}H_{34}O_{11}$	522.21	0.04	0.02
8.846	Rutin	$C_{27}H_{30}O_{16}$	610.153	0.10	0.06
8.853	(-)-Epicatechin gallate	$C_{22}H_{18}O_{10}$	442.09	0.02	0.23
8.853	Chalconaringenin	$C_{15}H_{12}O_5$	272.068	-	0.03
8.864	NP-018730	$C_{21}H_{20}O_{10}$	432.105	1.89	2.09
8.904	Trichbenzoisochromen A	$C_{14}H_{12}O_5$	260.068	-	0.02
8.924	Astilbin	$C_{21}H_{22}O_{11}$	450.116	0.01	0.01
8.954	Quercetin-3β-D-glucoside	$C_{21}H_{20}O_{12}$	464.095	0.08	0.09
8.987	(1S,3R,4R,6R)-4-Hydroxy-2,2,6-trimethyl-1-[(1E)-3-oxo-1-buten-1-yl]-7-oxabicyclo[4.1.0]hept-3-yl beta-D-glucopyranoside	$C_{19}H_{30}O_9$	402.189	0.06	0.04
9.014	Borapetoside A	$C_{26}H_{34}O_{12}$	538.205	0.02	-
9.036	Orientin 2''-O-gallate	$C_{28}H_{24}O_{15}$	600.112	0.04	-
9.056	Cynaroside	$C_{21}H_{20}O_{11}$	448.1	0.03	0.04
9.064	(3E)-2-Hydroxy-4-[(1S)-1-hydroxy-2,6,6-trimethyl-4-oxo-2-cyclohexen-1-yl]-3-buten-1-yl beta-D-glucopyranoside	$C_{19}H_{30}O_9$	402.189	0.11	-
9.269	5,7-Dihydroxy-2-(4-hydroxyphenyl)-4-oxo-4H-chromen-3-yl 6-O-(6-deoxyhexopyranosyl)hexopyranoside	$C_{27}H_{30}O_{15}$	594.159	-	0.03

9.332	Glucose 1-phosphate	$C_6 H_{13} O_9 P$	260.032		0.02
9.334	Coatline A	C21 H24 O_{10}	436.137	0.02	0.03
9.342	(2S,3S)-4-Hydroxy-2,3-bis(4-hydroxy-3-methoxybenzyl)butyl beta-L-glucopyranoside	$C_{26} H_{36} O_{11}$	524.226	-	0.02
9.365	Quercetin 3- (6''-p-hydroxybenzoylgalactoside)	$C_{28} H_{24} O_{14}$	584.116	0.22	0.28
9.378	Gaxilose	$C_{11} H_{20} O_{10}$	312.105	-	0.02
9.412	Aureusidin 6-glucuronide	$C_{21} H_{18} O_{12}$	462.08	0.03	0.02
9.443	Astragalin	$C_{21} H_{20} O_{11}$	448.101	0.07	-
9.5	Pelargonidin	$C_{15} H_{10} O_5$	270.053	0.02	0.02
9.544	NP-005166	$C_{30} H_{28} O_6$	484.195	0.04	0.03
9.616	Genistin	$C_{21} H_{20} O_{10}$	432.105	-	0.01
9.638	Azelaic acid	$C_9 H_{16} O_4$	188.105	0.07	0.07
9.73	1,3,4,6-Tetra-O-acetyl-2-deoxy-2-[(3-oxohexanoyl)amino]-beta-D-glucopyranose	$C_{20} H_{29} N O_{11}$	459.174	0.02	-
9.73	6-O-[(2E)-3-Phenyl-2-propenoyl]-1-O-(3,4,5-trihydroxybenzoyl)-β-D-glucopyranose	$C_{22} H_{22} O_{11}$	462.116	T	0.01
9.748	4-Allyl-2-methoxyphenyl 6-O-beta-D-xylopyranosyl-beta-D-glucopyranoside	$C_{21} H_{30} O_{11}$	458.179	0.01	0.01
9.766	Ceramidastin	$C_{26} H_{34} O_{11}$	522.21	0.12	0.11
9.826	Aurasperone C	$C_{31} H_{28} O_{12}$	592.158	0.01	0.01
9.837	4-Hydroxy-3-(3-methyl-2-buten-1-yl)phenyl 6-O-[(2R,3R,4R)-3,4-dihydroxy-4-(hydroxymethyl)tetrahydro-2-furanyl]-beta-D-glucopyranoside	$C_{22} H_{32} O_{11}$	494.176	0.02	T
9.948	Sulfurein	$C_{21} H_{20} O_{10}$	432.106	T	0.02
9.967	Indigotide B	$C_{22} H_{26} O_{10}$	450.152	-	0.01
10.032	Oxirapentyn B	$C_{18} H_{22} O_6$	334.141	0.03	0.11
10.052	3-(4-[1,3-Dihydroxy-1-(4-hydroxy-3-methoxyphenyl)-2-propanyl]oxy}-3-methoxyphenyl)propyl 6-deoxy-alpha-L-mannopyranoside	$C_{26} H_{36} O_{11}$	524.226	0.03	0.03
10.224	Vitexin 2''-p-hydroxybenzoate	$C_{28} H_{24} O_{12}$	552.127	0.01	0.02
10.251	Gallicynoic acid F	$C_{18} H_{32} O_6$	344.22	-	0.04
10.27	NP-015285	$C_{21} H_{20} O_9$	416.111	0.04	0.02
10.484	NP-018731	$C_{22} H_{22} O_{10}$	446.121	0.02	0.02

10.485	Eriodictyol	$C_{15} H_{12} O_{6}$	288.063	0.02	0.04
10.627	Luteolin	$C_{15} H_{10} O_{6}$	286.048	0.02	0.05
10.67	Dichotosinin	$C_{24} H_{30} O_{10}$	478.184	0.04	-
10.701	Sinapinic acid	$C_{11} H_{12} O_{5}$	206.058	T	0.01
10.852	Vitexin 2''-O- (E) -ferulate	$C_{31} H_{28} O_{13}$	608.153	-	0.03
10.867	Monascusone B	$C_{17} H_{18} O_{5}$	302.115	0.04	0.01
11.02	Glycitin	$C_{22} H_{22} O_{10}$	446.121	-	0.01
11.196	Vineomycin E	$C_{31} H_{34} O_{12}$	598.205	0.02	0.01
11.398	Apigenin	$C_{15} H_{10} O_{5}$	270.053	T	0.02
11.554	Corchorifatty acid F	$C_{18} H_{32} O_{5}$	328.225	0.25	0.2
11.688	NP-004917	$C_{15} H_{26} O_{3}$	276.17	0.01	0.01
11.7	Methoxyvestitol	$C_{17} H_{18} O_{5}$	302.115	0.04	-
11.805	Metatacarboline F	$C_{25} H2_{8} N_{4} O_{6}$	480.2	0.01	0.04
11.816	Virescenoside R3	$C_{33} H_{54} O_{13}$	658.356	0.02	-
12.222	Formononetin	$C_{16} H_{12} O_{4}$	268.073	0.02	0.21
12.258	6-O-(6-Deoxy-2-O-[(2E)-3-phenyl-2-propenoyl]-α-L-mannopyranosyl}-1-O-[(2E)-3-phenyl-2-propenoyl]-β-D-glucopyranose	$C_{30} H_{34} O_{12}$	632.21	T	0.02
12.392	Pinocembrin	$C_{15} H_{12} O_{4}$	256.074	-	0.01
12.543	Kurasoin A	$C_{16} H_{16} O_{3}$	256.11	0.01	0.05
12.644	Gallicynoic acid E	$C_{18} H_{30} O_{5}$	326.209	0.01	-
14.875	(-) -Sativan	$C_{17} H_{18} O_{4}$	286.12	-	0.04
15.403	NP-005013	$C_{16} H_{12} O_{5}$	284.068	-	0.03
18.031	Strobopinin	$C_{16} H_{14} O_{4}$	270.089	T	0.10
19.609	Palmitoleic Acid	$C_{16} H_{30} O_{2}$	276.209	0.02	-

Compounds less than 0.01% of the total area were considered as trace amounts and denoted as T.
